# ICTV Virus Taxonomy Profile: *Solinviviridae*

**DOI:** 10.1099/jgv.0.001242

**Published:** 2019-03-05

**Authors:** Katherine Brown, Ingrida Olendraite, Steven M. Valles, Andrew E. Firth, Yanping Chen, Diego M. A. Guérin, Yoshifumi Hashimoto, Salvador Herrero, Joachim R. de Miranda, Eugene Ryabov, ICTV Report Consortium

**Affiliations:** ^1^​ Department of Pathology, University of Cambridge, Cambridge CB2 1QP, UK; ^2^​ United States Department of Agriculture, Agricultural Research Service, Gainesville FL 32608, USA; ^3^​ United States Department of Agriculture, Agricultural Research Service, Beltsville MD 20705, USA; ^4^​ Department of Biochemistry and Molecular Biology, University of the Basque Country (EHU), Biophysics Institute (CSIC-UPV/EHU), Bº Sarriena S/N, 48940 Leioa, Spain; ^5^​ BioMarin Pharmaceutical Inc., San Rafael CA 94901, USA; ^6^​ Department of Genetics, Universitat de València, Burjassot, Spain; ^7^​ Department of Ecology, Swedish University of Agricultural Sciences, Uppsala 750 07, Sweden

**Keywords:** *Soliniviviridae*, ICTV Report, taxonomy

## Abstract

*Solinviviridae* is a family of picorna/calici-like viruses with non-segmented, linear, positive-sense RNA genomes of approximately 10–11 kb. Unusually, their capsid proteins are encoded towards the 3′-end of the genome where they can be expressed both from a subgenomic RNA and as an extension of the replication (picorna-like helicase–protease–polymerase) polyprotein. Members of two species within the family infect ants, but related unclassified virus sequences derive from a large variety of insects and other arthropods. This is a summary of the International Committee on Taxonomy of Viruses (ICTV) Report on the *Solinviviridae*, which is available at www.ictv.global/report/solinviviridae.

## Abbreviations

FSD, frameshift domain; RdRP, RNA-dependent RNA polymerase; VP1, viral protein 1; VPg, viral protein genome-linked.

## Virion

**Fig. 1.**
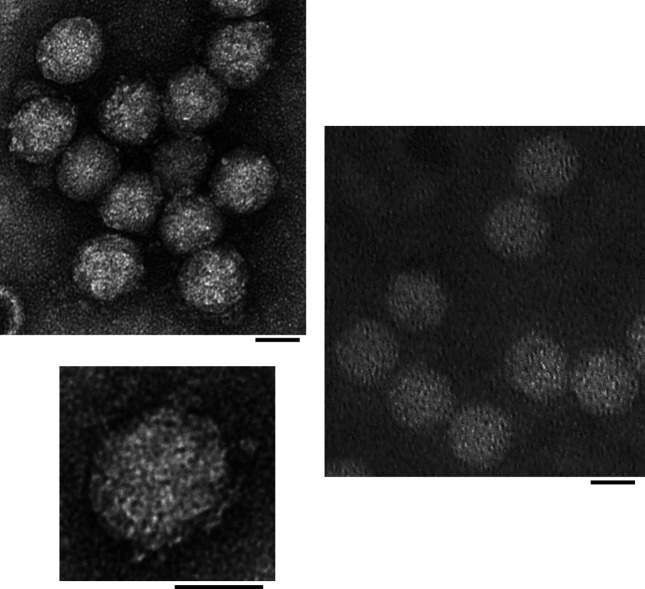
Electron micrographs of negatively-stained Solenopsis invicta virus 3 (left and inset below) and Nylanderia fulva virus 1 (right) particles (scale bars, 20 nm) [reproduced from [1, 2]; U.S. government material as public domain content].

Solenopsis invicta virus 3 (genus *Invictavirus*) and Nylanderia fulva virus 1 (genus *Nyfulvavirus*) particles are icosahedral, with a diameter of 26–30 nm and have apparent projections (Table 1) (Fig. 1) [1, 2]. A similar morphology has been reported for the related unclassified kelp fly virus where the projections – present at some but not all fivefold axes – protrude approximately 6 nm above the surface of the 33 nm-diameter virion [3, 4]. Solenopsis invicta virus 3 virions contain a major component, viral protein 1 (VP1), and a minor component, VP1-FSD, where FSD (frame shift domain) is an extension domain that is appended to a proportion of VP1 proteins via ribosomal frameshifting [5]. VP1 is thought to form the virion shell and FSD the projections. A further virion protein, VP2, has been detected only at substoichiometric levels. Particles are presumed to be T=3 containing 180 copies of VP1 [5].

**Table 1.** Characteristics of members of the family *Solinviviridae*

Typical member: Solenopsis invicta virus 3, DM (FJ528584), species *Solenopsis invicta virus 3*, genus *Invictavirus*	
Virion	Non-enveloped, 26–30 nm diameter with apparent projections
Genome	10–11 kb of positive-sense, non-segmented RNA
Replication	Not studied; presumed to be similar to other picorna/calici-like viruses
Translation	From genomic RNA (replication and capsid proteins) and subgenomic RNA (capsid proteins)
Host range	Arthropoda
Taxonomy	Includes the genera *Invictavirus* and *Nyfulvavirus*

## Genome

**Fig. 2.**
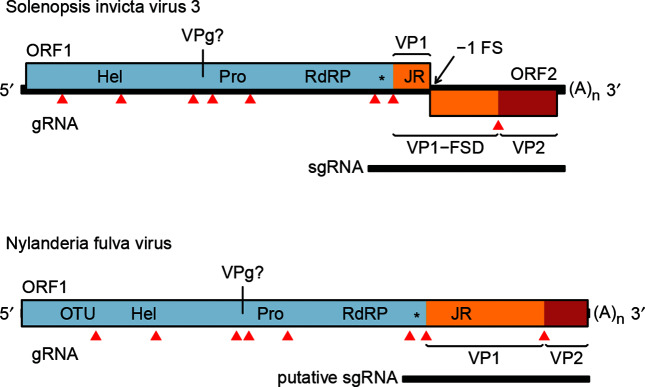
Genome organization of Solenopsis invicta virus 3 and Nylanderia fulva virus 1. Predicted protein domains are indicated as follows: Hel – helicase, Pro – protease, RdRP – RNA-dependent RNA polymerase, JR – jelly-roll fold capsid, * – dsRNA binding protein (potentially a suppressor of host RNAi), OTU – ovarian tumor domain. Mapped virion proteins in Solenopsis invicta virus 3 are coloured as follows VP1 and VP1-FSD – orange, VP2 – red (VP=viral protein, FSD=frameshift domain). Predicted cleavage sites are indicated by red triangles. The genome is polyadenylated and believed to have a viral protein genome-linked (VPg) covalently attached to the 5′- end.

The genome comprises a single, positive-sense RNA molecule of 10–11 kb with a 3′-poly(A) tail and is presumed to have a viral protein genome-linked (VPg) covalently attached to the 5′-end [1]. The genome of Solenopsis invicta virus 3 contains a long open reading frame (ORF) covering approximately three-quarters of the genome, encoding helicase, protease and RNA-dependent RNA polymerase (RdRP) domains followed by the jelly-roll fold capsid protein (VP1) (Fig. 2). The ORF1 polyprotein is presumably cleaved by the virus 3C-like protease. A dsRNA-binding domain with homology to the 1A suppressor of silencing protein of Drosophila C virus (family *Dicistroviridae*) is present between the RdRP and jelly-roll fold domains. Additional proteins are probably encoded upstream of helicase (one protein) and between helicase and protease (two proteins, one presumed to be a VPg) [2]. ORF2 is expressed via ribosomal frameshifting leading to an ORF1–ORF2 polyprotein. The 5′- and 3′-halves of ORF2 encode FSD and VP2, respectively [5]. A subgenomic RNA encoding the dsRNA-binding protein, VP1, FSD and VP2 is produced [5]. Nylanderia fulva virus 1 has a single, long ORF encoding similar domains to the Solenopsis invicta virus 3 ORF1–ORF2 fusion and in the same order [2]. Related virus sequences have similar helicase–protease–polymerase–jellyroll domain organization [2].

## Replication

Replication is assumed to be similar to other picorna/calici-like viruses.

## Taxonomy

The family *Solinviviridae* includes two genera: *Invictavirus* and *Nyfulvavirus*. Similar to picorna/calici-like viruses, solinivivirus genomes encode a superfamily III helicase, a 3C-like chymotrypsin-related cysteine protease and a superfamily I RdRP. In contrast to members of the order *Picornavirales*, but similar to members of the family *Caliciviridae*, there is only a single jelly-roll fold capsid domain. Encoding of capsid proteins at the 3′-end of the genome, production of a capsid-encoding subgenomic RNA and the presence of a capsid projection domain are also calicivirus-like features [5]. Demarcation of *Solinviviridae* genera is based on phylogenetic divergence; however, formal genus demarcation criteria have not yet been established. Members of two species – *Solenopsis invicta virus 3* and *Nylanderia fulva virus 1* – infect ants; however, related unclassified virus sequences have been isolated from a variety of other insects and other arthropods [2, 6].

## Resources

Full ICTV Report on the family *Solinviviridae*: www.ictv.global/report/solinviviridae.
